# Editorial: COVID-19 and eating disorders 2023: lessons learnt and future directions for research

**DOI:** 10.3389/fpsyt.2024.1453218

**Published:** 2024-07-05

**Authors:** Paolo Meneguzzo

**Affiliations:** ^1^ Department of Neuroscience, University of Padova, Padova, Italy; ^2^ Padova Neuroscience Center, University of Padova, Padova, Italy

**Keywords:** eating disorder, health care, COVID - 19, pandemic, qualitative study, weight disorders, therapeutic alliance

The recent COVID-19 pandemic has been likened to a stormy sea in the field of eating disorders (ED), with profound effects on health status (1). The pandemic has exacerbated the presence of symptoms of ED throughout the entire spectrum of the disorder, leading to changes in weight and elevated levels of anxiety and depression, which appear to be correlated with lockdown measures (2). Indeed, lockdowns have presented unique challenges that may have disproportionately impacted individuals with EDs (3), as well as posing difficulties for their siblings (4). Recent literature published during the pandemic has suggested the presence of possible changes in the psychological presentation of these disorders (5, 6), with the general population reporting higher levels of emotional eating and binge disorders (7). However, mitigation of the pandemic effects appears to have reduced the effects of the alarm pressure that emerged a few years ago and called for time-sensitive interventions (8). Furthermore, the increased demand for intensive care treatment for EDs has underscored the urgent need for adequate capacity and professionals involved in ED healthcare (9, 10), but this seems to be challenging in different places and sociocultural aspects (11).

For these reasons, it is crucial to raise awareness of the significant insights the pandemic has revealed globally in the field of eating disorders. The articles featured in this Research Topic, encompassing a diverse range of research designs, methodologies, and theoretical frameworks, illustrate the breadth of the literature exploring the intersection of EDs and the recent COVID-19 pandemic.

Napp et al. discovered an increase in disordered eating among boys in Germany after the pandemic, particularly in younger boys, despite lower overall prevalence rates compared to before 2020. Differences were found in prevalence, which was higher among girls and older participants. Anxiety and depressive symptoms were associated with an increased probability of ED, consistent with the literature, although specific gender differences suggested greater vulnerability in girls to anxiety. Moreover, only gender, depression, and anxiety symptoms were associated with ED symptoms 1.5 years after the onset of the pandemic, highlighting once again the global effects on young people’s life trajectories. Finally, these findings stress the importance of ongoing research, prevention, and intervention programs that address age- and gender-specific differences and the need for adapted screening tools for youth EDs.

Elran-Barak et al. examined the therapeutic alliance in multidisciplinary ED treatment during the COVID-19 pandemic, focusing on patient-dietician and patient-psychotherapist dyads. They discovered a stronger concordance in the patient-dietician dyads compared to the patient-psychotherapist dyads. Furthermore, they found that severe eating psychopathology was associated with a weaker therapeutic alliance, while general psychopathology in patients was associated with a weaker alliance with dieticians. This pilot study, involving 63 patients and their psychotherapists and dieticians surveyed during the pandemic, used the Working Alliance Inventory (WAI-S) to assess therapeutic alliance. The study underscores the need for more longitudinal research to validate these findings and explore the impact of multidisciplinary therapeutic alliances on treatment outcomes in online ED treatment settings during the pandemic and beyond.

Schraml et al. evaluated differences in quality of life, symptoms of depression, anxiety, and ED in two different groups of people with severe obesity in a multimodal behavioral weight loss program, initiated before and during the pandemic. The study evaluated the possible difference between a face-to-face and a virtual intervention. They found no differences in depression, anxiety, and perceived stress, nor a reduction in body weight in 67% of the participants or a stabilization in 16% of them. The virtual intervention helped stabilize and improve the physiological and psychological burdens of people with severe obesity during the uncertain time of the pandemic.

Boltri et al. conducted a systematic review of the literature on the short- and long-term effects of the pandemic on care provider systems, looking for possible evidence that could help adapt care programs to the changes that emerged during the pandemic. They found that the pandemic caused a sudden and significant increase in hospital visits and admissions among children and adolescents. Despite a decrease during the second year of these numbers, there are still higher requests than before the pandemic, with new obstacles to possible access to care treatment. As a result, healthcare providers are now developing innovative interim strategies and multidisciplinary telehealth solutions to address these challenges.

Marphy-Morgan et al. performed a mixed methods approach to explore lived experiences of remote care for EDs during the pandemic. This study, involving 211 people with ED through interviews and surveys, found that ED symptoms worsened during the pandemic due to isolation, lack of routine, and negative emotions. Remote care provided flexibility and social connection, but faced barriers such as lack of awareness, digital access, and competing commitments. The challenges included the overly clinical nature of some approaches, quality concerns, and distress from self-view on video calls. The participants recommended more holistic and hybrid care options, incorporating real recovery stories and digital literacy training.

The current Research Topic has generated a diverse collection of studies on the effects observed in EDs during the pandemic, ranging from the challenges to potential future research directions. New technologies, novel approaches, and the need for gender- and age-specific tools and methodologies stand out as the most significant elements that the field of EDs should consider as guiding lights. Finally, if the field of impact of COVID-19 on eating disorders could be depicted as a stormy sea, we could see a lighthouse in this picture, standing tall among turbulent waves. The stormy sea represents the challenges and disruptions caused by the pandemic, including increased isolation, anxiety, and disruptions to routines. Amidst this turmoil, the lighthouse symbolizes resilience, guidance, and hope, fundamental elements in the ED field.

## Author contributions

PM: Writing – original draft.
